# Advances in oncolytic virotherapy

**DOI:** 10.1038/s43856-022-00098-4

**Published:** 2022-04-07

**Authors:** Stephen J. Russell, John C. Bell, Christine E. Engeland, Grant McFadden

**Affiliations:** 1 Mayo Clinic, Rochester, MN USA; 2 Vyriad, Rochester, MN USA; 3 Ottawa Hospital Research Institute, University of Ottawa, Ottawa, ON Canada; 4 Clinical Cooperation Unit Virotherapy, German Cancer Research Center (DKFZ) and National Center for Tumor Diseases (NCT), Heidelberg, Germany; 5 Department of Medical Oncology, University Hospital Heidelberg, Heidelberg, Germany; 6 Faculty of Health/School of Medicine, Institute of Virology and Microbiology, Center for Biomedical Education and Research (ZBAF), Witten/Herdecke University, Witten, Germany; 7 Center for Immunotherapy, Vaccines, and Virotherapy (CIVV), Biodesign Institute, Arizona State University, Tempe, AZ USA; 8 OncoMyx Therapeutics, Phoenix, AZ USA

## Abstract

Recent years have seen rapid advances in the preclinical development and clinical evaluation of oncolytic (cancer-lysing) virus-based therapies, and these are emerging as treatment modality for some cancers. There are challenges to address, however, if we are to maximize the impact of these therapies in patients.

In this Viewpoint, four experts in the oncolytic virotherapy field outline some of the important advances in this area, highlight key challenges that still need to be addressed to move these therapies into the clinic, and outline areas of research to watch. These range from from genetic engineering and manufacturing of safe and effiacious therapeutic viruses, to testing in animal models and ultimately in patients. Key themes that emerge are the need to optimize these therapies to modulate the tumor immune microenvironment and the potential for combining virotherapy with other immunotherapies to derive maximal clinical benefit.

## Stephen J. Russell

**Figure:**
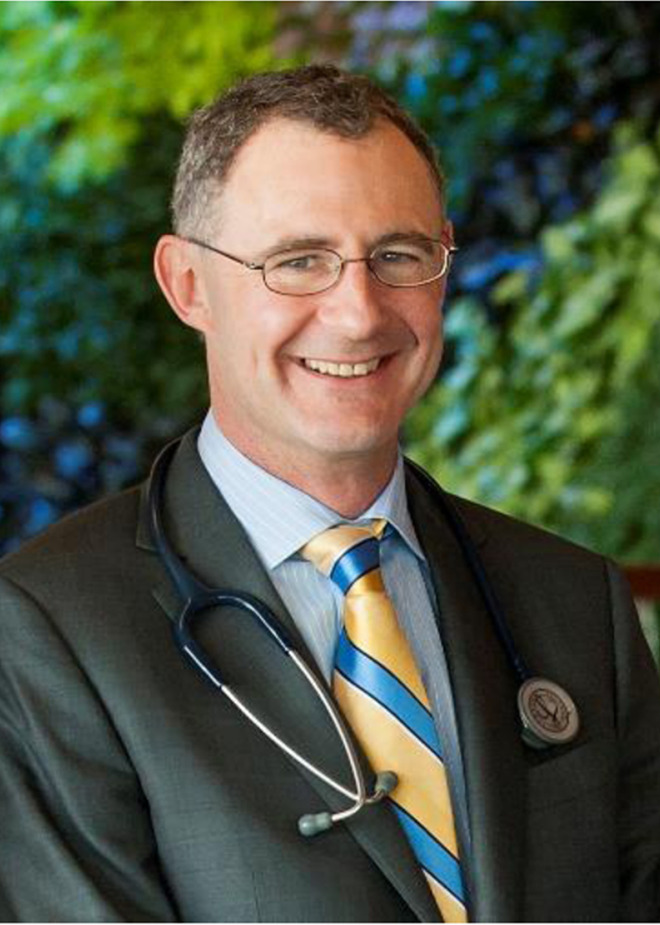
Mayo Clinic

Oncolytic viruses (OVs) are set to become the third leg of the cancer immunotherapy stool, functioning as antigen-agnostic vaccines to boost the activity of checkpoint blockade and adoptive cell therapies. They kill tumor cells in situ, inflame the tumor, recruit antigen-presenting cells which phagocytose tumor antigens, and drive the amplification of antitumor T cells, even though the identity of the tumor antigens is unknown.

Preclinically, the variety of novel OV platforms, transgene payloads and combination therapies under investigation is burgeoning. Emerging themes are that genetic payloads such as interleukin-12 and anti-CTLA4 can powerfully increase the immune-boosting activity of several OVs. On the targeting front, entry specificities of certain OV platforms can now be precisely controlled by engineering their surface attachment proteins to display tumor-targeting ligands such as single chain antibodies. Unwanted OV tropisms that are potentially harmful to normal tissues can also be efficiently removed by engineering microRNA target sequences into OV genomes (microRNA targeting). As for combination therapies, OV potency has been increased by rational partnering with drugs that increase intratumoral virus spread by suppressing immune defences. There is also promise in combinations with drugs that boost immune control of the tumor by inhibiting immune checkpoints, or with CAR T cells that can carry OVs into tumors where, in turn, the delivered OV can convert the tumor into a more susceptible CAR T target. These observations provide a compelling case that for OVs to be maximally effective in the clinic they will have to be incorporated into rational combination regimens.

Clinically, there has been steady progress in the field subsequent to the original FDA approval of Amgen’s intratumoral Herpes Simplex Virus (HSV)-based talimogene laherparepvec (T-VEC) therapy for advanced melanoma in 2015. One encouraging recent development is that teserpaturev, a Daiichi Sankyo-developed HSV, was granted provisional regulatory approval in Japan for stereotactic intratumoral therapy of patients with inoperable glioma. The approval was based on a small non-randomized study in which 12 of 13 patients treated with oncolytic virus were still alive at the one-year landmark, an outstanding result considering the expected 7-month survival for this group of patients with recurrent glioblastoma. Replimune’s new HSV constructs encoding powerful immune-activating transgenes (IL-12 and an anti-CTLA4 antibody) are showing early promise even outside the realm of skin cancers, which are still the only cancers approved for OV therapy in the USA. However, in a recent setback Amgen’s randomized phase 3 melanoma trial comparing anti-PD1 pembrolizumab monotherapy versus T-VEC plus pembrolizumab was stopped early after an interim futility analysis. This was surprising, particularly in light of the large randomized phase 2 study previously conducted by Amgen which showed strong superiority of TVEC plus ipilumumab (anti-CTLA4) versus ipilumumab alone.

Compared to the intratumoral approach, clinical trials of intravenous OV therapy have lagged, in part because of the need to use viruses which cancer patients have not previously encountered and become immune, and that can be efficiently manufactured to facilitate high dose therapy. Fortunately, progress is now accelerating and results from several ongoing systemic OV therapy trials are eagerly awaited. One notable recent success, reported at the annual meeting of the American Society of Clinical Oncology in May 2021, was the demonstration of clear durable responses in patients with heavily pretreated hematologic malignancies in a phase 1 clinical trial of a single intravenous dose of Vyriad’s Voyager-V1, a recombinant vesicular stomatitis virus.

## John C. Bell

**Figure:**
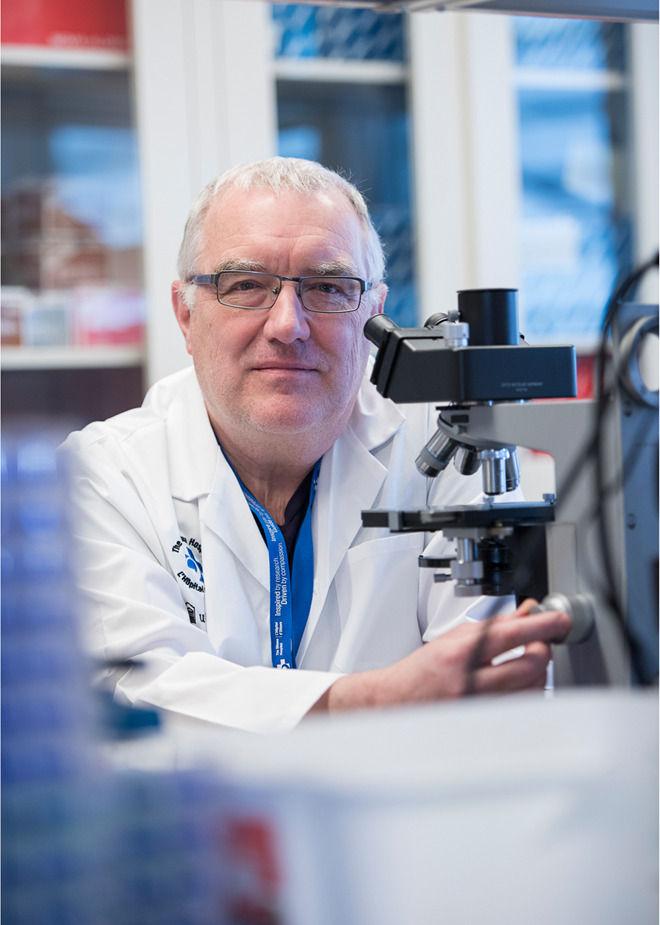
The Ottawa Hospital

It has become apparent over the last several decades of research and clinical testing that OVs have the potential to be much more than simply cytolytic agents and can therapeutically impact many of the hallmarks of cancer described by Hanahan and Weinberg. As certain OV platforms can be engineered to express novel therapeutic transgenes, they transition from being ‘one-trick ponies’ to complex biological machines that can catalyze the kinds of complex treatment protocols that are likely required to tackle heterogeneous cancers.

Beyond their direct effect on tumor cells, it may be that the real power of the OV is its ability to engage the patient’s own immune system, triggering it to recognize and destroy cancer cells. Large DNA viruses like herpesviruses and poxviruses are now being programmed to express multiple complementary immune-modulating genes with the goal of remodeling the tumor microenvironment and reversing pro-malignant, immune-suppressive milieus. As these OVs replicate and spread within and between tumors, along the way they can, in a targeted fashion, disseminate genetic information in the form of proteins, mRNAs or microRNAs. There is a dichotomy here in that viruses on their own encode multiple innate and adaptive immune-suppressing genes that are critical to their ability to at least temporarily avoid immune clearance. A balance of virally-encoded immune suppression is required to facilitate OV infection and spread while at the same time recruiting therapeutic immune cells to attack and destroy cancer cells. Perhaps the use of regulatable gene expression systems within OVs to tactically deploy payloads when and where they will be most effective will be critical for optimal use of this platform. Some inducible promoter systems for control of cell gene expression have entered clinical trials and it would seem reasonable to expect they could be adapted for some OV platforms.

As the immunotherapy field has evolved, many powerful cell-based therapeutics have been developed including, but not limited to, chimeric antigen receptor (CAR) T cells and ex vivo-expanded tumor-infiltrating lymphocyte products. Cell products clearly have single-agent activity in some indications. However, I would argue that to reach their full therapeutic potential combination with a strategically-designed OV may be key. Recruitment and sustained activation of cell products within the tumor can be facilitated by OV expression of novel immune-activating molecules. For example, virus expression of potent tailored versions of cytokines or chemokines tethered within the tumor microenvironment, localized production of T cell engagers and/or extracellular matrix degrading enzymes are all being tested as complementary strategies to enhance immune cell therapy.

## Christine E. Engeland

**Figure:**
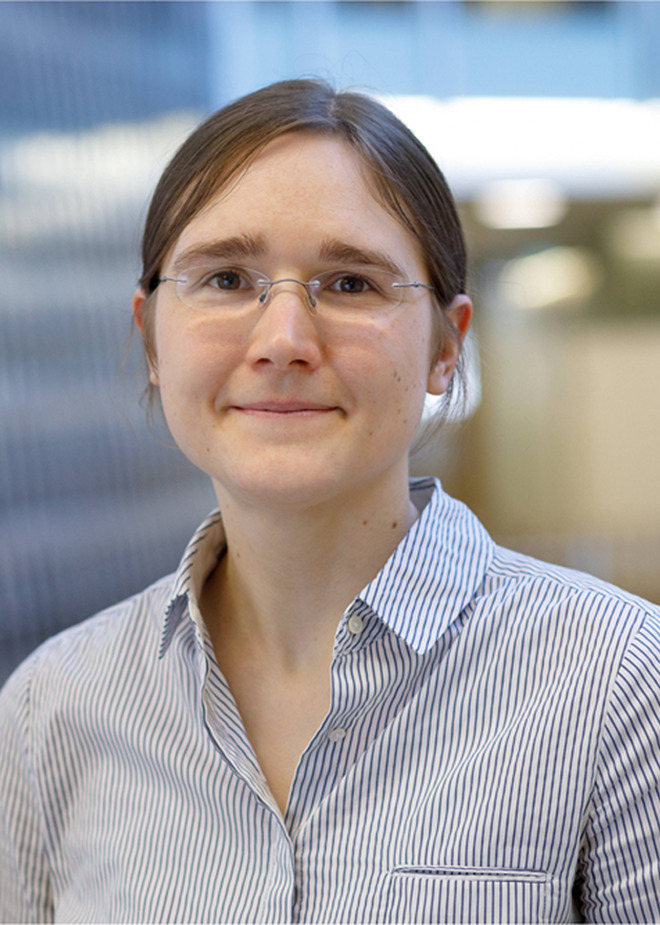
Philip Benjamin

From a theoretical standpoint, oncolytic virotherapy is a compelling approach: the therapeutic agent amplifies selectively within malignant cells, leads to tumor cell lysis and at the same time initiates antitumor immunity. Now we must demonstrate that this concept translates into clinical practice and tangible benefits for patients.

Although virotherapy as standalone treatment may be effective in certain situations, combination therapies are necessary to realize its full potential. Arguably, combination with immune checkpoint blockade is the most obvious approach, following the notion that virotherapy as an antigen-agnostic cancer vaccine elicits priming of tumor-reactive T cells, which are then disinhibited by checkpoint blockade. Consequently, a number of clinical trials are currently testing this approach. Although the recent  phase 3 trial of T-VEC and pembrolizumab failed to demonstrate improved outcome despite promising earlier data, further analyses will show which subgroups of patients can benefit from virotherapy combined with checkpoint blockade.

Early-phase trials of experimental therapies typically enroll advanced-stage, heavily pre-treated cancer patients. Lately, virotherapy has been applied in ‘window of opportunity’ studies, in a neoadjuvant or adjuvant setting. This is clinically feasible, for example in colorectal cancer, breast cancer, or glioma, and may be most effective in tumors with high neoantigen load. Indeed, these clinical situations may allow oncolytic vaccination to take full effect.

At present, diverse virus platforms are developed in parallel. These viruses differ in cellular pathways exploited for replication and immunogenicity. As head-to-head comparisons are challenging, biomarkers that predict which virus is most effective against which tumor are lacking. While defects in antiviral response pathways may predict sensitivity to a broad range of virotherapeutics, there are certainly disease- and virus-specific biomarkers that remain to be discovered.

Moreover, preclinical models often fail to recapitulate the biology, complexity and heterogeneity of human cancers. Therefore, even early phase clinical trials must incorporate translational research programs to decipher mechanisms of response as well as resistance. Insights gained from serial biopsies and immunomonitoring will also inform on rational combination approaches. New high dimensional analytic tools such as multiparameter flow cytometry, single-cell sequencing and proteomics should also be applied to unravel the complex interactions of oncolytic viruses within the tumor microenvironment.

The next few years will tell whether oncolytic virotherapy is to become an established modality of cancer immunotherapy.

## Grant McFadden

**Figure:**
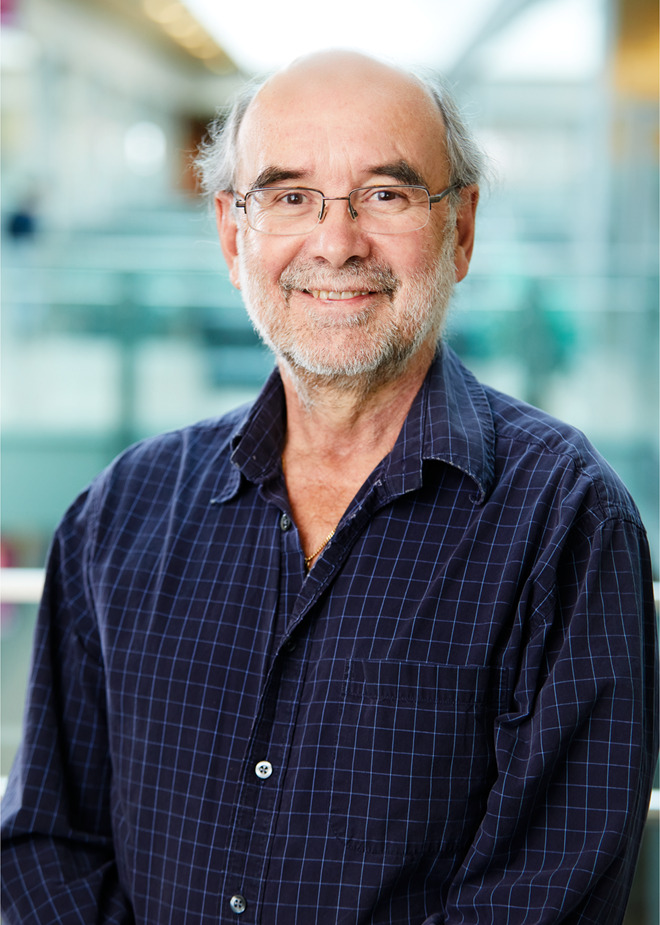
Brandon Sullivan

The observation that fever-inducing infections in cancer patients can occasionally lead to tumor regression was first made over a century ago. The exploitation of live OV therapy has been under detailed scrutiny for decades now, and the clinical experience has been generally one of excellent safety but variable clinical efficacy. Major strides have been made, for example in the appreciation that OVs can concurrently stimulate novel acquired cellular immune responses against tumor antigens while co-developing immunity to the OV itself. Below are six key challenges being addressed to develop OVs as useful clinical tools for the next generation of medical oncologists.

The first is the choice of virus platform. Many dozens of OV candidates are under development, ranging from small RNA viruses to large DNA viruses, but it is very difficult to compare one platform to another using patient data because clinical trial regimens and protocols vary widely. Instead, the key differentiating criteria relate to the virus biology, tumor tropism and the genetic payload. The second challenge is the selection of therapeutic transgene(s). The purpose of inserting transgenes in the virus genetic material is to upregulate acquired antitumor responses that continue long after the initiating virus has been cleared. There is no agreement on how many such transgenes are optimal for these tasks, but the variety of potential candidate genes include a long list of cytokines, enzymes, antibodies, immune cell engagers and biologics. The third is that mice really do lie about humans. The difficult truth is that the anticancer immune response from any test OV in tumor-bearing mice is poorly predictive of the orthologous response using the identical virus in cancer patients. Even immune-reconstituted humanized mice can only at best provide semi-chimeric tumor microenvironments that are imperfect surrogates of human tumors. The fourth challenge concerns virus delivery. Any OV can be delivered by direct intratumoral injection, but many human cancers mandate some form of systemic delivery. Multiple strategies are being investigated preclinically, for example the delivery of OVs to tumor beds using migratory carrier cells, but clinical studies are required to prove that any OV can be systemically delivered in patients. The fifth is clinical grade manufacturing. All OVs developed in academic labs exploit methods of virus propagation and purification that are not directly scalable for Good Manufacturing Practice conditions. The growing biologics-as-drugs biotech industry will hopefully solve most of these nontrivial technical issues. The sixth and final challenge is devising combinatorial therapies. Newer anticancer technologies like immune checkpoint inhibitors, targeted drugs and CAR T cells are rapidly becoming more entrenched in the clinical space, and it is critical to understand how best to combine them with OVs. Many currently intractable cancers tend to be immune-cold—that is, they are poorly infiltrated with reactive leukocytes—and, as such, patients become operationally immunodeficient against their own cancer. The goal of next-generation OVs is to promote immune infiltration, converting such tumors to an immune-hot state, and thereby generating more robust antitumor immunity to drive longer term cancer regression.

